# Clinical Effectiveness of Oral Semaglutide in Women with Type 2 Diabetes: A Nationwide, Multicentre, Retrospective, Observational Study (Women_ENDO2S-RWD Substudy)

**DOI:** 10.3390/nu17142349

**Published:** 2025-07-17

**Authors:** Rebeca Reyes-Garcia, Oscar Moreno-Pérez, Cristina Guillen-Morote, Inés Modrego-Pardo, Viyey Kishore Doulatram-Gamgaram, Carlos Casado Cases, Nieves Arias Mendoza, Cristina Tejera-Pérez, Jersy Cárdenas-Salas, Sandra Martínez-Fuster, Beatriz Lardiés-Sánchez, Rosa Márquez-Pardo, Pedro Pinés, Antonio Tejera-Muñoz, José Carlos Fernández-García, on behalf of the SEEN Diabetes Area

**Affiliations:** 1 Endocrinology Unit, University Hospital of Torrecárdenas, 04009 Almería, Spain; 2 CIBER de Fragilidad y Envejecimiento Saludable “CIBERFES”, Instituto de Salud Carlos III, 28029 Madrid, Spain; 3 Department of Endocrinology and Nutrition, General University Hospital Dr. Balmis of Alicante, Institute of Health and Biomedical Research of Alicante (ISABIAL), 03010 Alicante, Spain; omorenoperez@hotmail.es (O.M.-P.); guillen.cristina1996@gmail.com (C.G.-M.); antoniotemu@gmail.com (A.T.-M.); 4 Department of Clinical Medicine, Miguel Hernández University, 03550 Elche, Spain; 5 Department of Endocrinology and Nutrition, Marina Baixa Hospital, 03570 Villajoyosa, Spain; inesmodregopardo@gmail.com; 6 Department of Endocrinology and Nutrition, Regional University Hospital of Malaga, 29010 Málaga, Spain; viyu90@hotmail.es (V.K.D.-G.); jcfernandez@uma.es (J.C.F.-G.); 7 Biomedical Research Institute of Malaga (IBIMA), Faculty of Medicine, University of Malaga, 29010 Málaga, Spain; 8 Department of Endocrinology and Nutrition, University Hospital Fundación Jiménez Díaz, 28040 Madrid, Spainjersy_cardenas@hotmail.com (J.C.-S.); 9 Department of Endocrinology and Nutrition, General University Hospital of Elda, 03600 Alicante, Spain; nieves@socue.es (N.A.M.); sanmarfus@hotmail.com (S.M.-F.); 10 Department of Endocrinology and Nutrition, University Hospital Complex of Ferrol, 15405 A Coruña, Spain; cristinatejera.mui@gmail.com; 11 Department of Endocrinology and Nutrition, Obispo Polanco Hospital, 44002 Teruel, Spain; bealardies@gmail.com; 12 Department of Endocrinology and Nutrition, Hospital Juan Ramón Jiménez, 21005 Huelva, Spain; rosa_marquez_pardo@hotmail.com; 13 Department of Endocrinology and Nutrition, Albacete University Hospital Complex, 02006 Albacete, Spain; ppines77@hotmail.com; 14 Health Science Faculty-HM Hospitals, Camilo José Cela University, 28692 Madrid, Spain; 15 HM Hospitals Health Research Institute, 28015 Madrid, Spain; 16 Centro de Investigación Biomédica en Red de Diabetes y Enfermedades Metabólicas, Asociadas (CIBERDEM), Instituto de Salud Carlos III, 28029 Madrid, Spain

**Keywords:** real life, women, GLP-1 Agonist, effectiveness, cardiovascular disease

## Abstract

**Background:** Sex differences in type 2 diabetes (T2D) are a growing area of diabetes research. No data have been reported on sex differences with oral semaglutide (oSEMA) in a real-world setting. **Methods**: We included people with T2D who started treatment with oSEMA in routine clinical practice between November 2021 and November 2022, with at least one report of clinical follow-up (FU) data at 3 months. We evaluated in women with T2D (WWT2D) the clinical effectiveness of oSEMA and factors associated with clinical response and persistence. We also analyzed differences in baseline characteristics, clinical effectiveness, persistence rates and safety according to biological sex. **Results**: Of the 1018 subjects [median age: 63 years, body mass index (BMI): 33.8 kg/m^2^, HbA1c: 7.8%], 469 were WWT2D. In WWT2D, oSEMA reduced HbA1c by 0.7% [−0.1 to −1.3] and 0.9% [−0.2 to −1.5] at the 6- and 12-month FU visits, while weight decreased by 4.6% [2.0 to 7.9] and 7.2% [2.5 to 10.9], respectively. Weight loss was >10% in 29.8% of WWT2D (95% CI 25.8 to 34.1); meanwhile, the combined endpoint (HbA1c decrease ≥ 1% + weight reduction ≥ 5%) was achieved in 23.5% (95% CI 19.8 to 27.5%) of WWT2D at the 12-month FU visit. Achievement of glycaemic targets was similar in women and men (59.3% vs. 61.1%). We found no sex differences in weight loss (6.9% vs. 6.8%), oSEMA maintenance dose, persistence rate (76.3% vs. 77.3%), or adverse events. **Conclusions**: oSEMA was effective and safe in WWT2D in a real-world setting, with nearly one-third of patients reporting weight loss >10% and more than two-thirds achieving HbA1c < 7%. oSEMA showed no sex bias in terms of effectiveness and safety.

## 1. Introduction

While the prevalence, incidence and burden on healthcare systems of type 2 diabetes (T2D) are steadily increasing worldwide, potential sex differences are a growing aspect of diabetes research, as we move towards the development and standardization of precision medicine [1]. In line with this, it has been shown that women with T2D (WWT2D), in comparison with men, have an increased risk of cardiovascular disease (CVD) and mortality, and that psychosocial risk factors have a greater impact on the development of CVD and T2D [1].

Likewise, large biological sex differences in diabetes treatment have also been reported. Thus, young WWT2D are less likely to receive guideline-recommended CVD treatment than men [2]. Moreover, cardiovascular outcome trials have reported a lower use of statins, aspirin and beta-blockers in WWT2D, despite a higher prevalence of history of stroke and heart failure [3]. Regarding cardioprotective glucose-lowering medications, a Danish cohort study reported that sodium–glucose cotransporter-2 inhibitors (SGLT2is) or GLP-1 receptor agonists (GLP-1 RAs) were less commonly prescribed in WWT2D and CVD [4]. In addition, medication adherence has been reported to be lower in women than in men.

On the other hand, sex differences in the clinical efficacy of GLP-1RAs have also been described. Thus, cumulative evidence suggests that women have greater weight loss with GLP-1 RAs [5,6], but also exhibit a greater risk of gastrointestinal side effects [7]. Regarding glycemic benefit, despite similar HbA1c reductions with different GLP-1 RAs have been reported in clinical trials between both sexes [6,8], in an analysis of real-world data, women had a greater glycaemic benefit with GLP-1 RAs [9].

To our knowledge, there is no data on potential sex differences in people treated with oral semaglutide in a real-world setting. Therefore, in this study, we evaluated the clinical efficacy of oral semaglutide in women, and assessed factors associated with clinical response and persistence in this population, addressing sex differences.

## 2. Materials and Methods

### 2.1. Study Design and Participants

This is a substudy of the ENDO2S-RWD, a real-world, nationwide, multicentre, retrospective, observational study conducted to evaluate the effectiveness and safety of oral semaglutide in people with T2D [10]. In the ENDO2S-RWD study, we enrolled people living with type 2 diabetes (PLWT2D), aged 18 years or over, who had initiated oral semaglutide according to the clinician’s criteria in routine clinical practice. PLWT2D from 12 health centers within the Spanish National Health System were included if they had at least one report of clinical follow-up (FU) data after three months, between November 2021 and November 2022 [10]. Clinical outcomes were recorded in two periods: between 3 and 6 months (6-month FU visit) and between 6 and 12 months (12-month FU visit). Baseline characteristics, persistence, reasons for discontinuation and safety data were assessed for the entire cohort. In contrast, the clinical endpoints were only assessed for PLWT2D who continued oral semaglutide. Clinical data were manually extracted from the digital medical records. The primary endpoint was defined as a weight loss of ≥5% from the baseline weight and an HbA1c reduction of ≥1% at 6 and 12 months FU. The secondary endpoints were changes in HbA1c (%) and weight loss reduction [kg and weight loss percentage (WLP)] from baseline to 6 and 12 months FU. We assessed drug safety, factors associated with response, drug persistence and major cardiovascular adverse events (MACEs).

In this substudy (Women_ENDO2S-RWD Substudy), our aims were to evaluate the clinical effectiveness of oral semaglutide and its associated factors in WWT2D, and to evaluate potential differences in baseline characteristics according to sex. We also examined differences in the achievement of clinical goals between women and men.

This study was endorsed by the Spanish Society of Endocrinology and Nutrition and approved by the Ethics Committee of Dr Balmis General University Hospital (Alicante, Spain) (2022-0386).

### 2.2. Statistical Analysis

The Wilcoxon signed-rank test for paired samples assessed the differences in continuous variables. Multiple logistic regression models were constructed to explore the association between baseline data and clinical outcomes at short and medium term, estimating odds ratios [95% confidence interval (CI)]. All tests were two-tailed, and statistical significance was set at *p* < 0.05. IBM SPSS Statistics 25 software was used for analyses.

## 3. Results

Of the 1018 PWT2D in the global cohort, 469 were women [median age: 63 years, body mass index (BMI): 34.4 kg/m^2^, HbA1c: 7.7%, median diabetes duration 7 years] with a median semaglutide treatment duration of 19 weeks (15 to 25) at the 6-month FU visit and 45 weeks (36 to 52) at the 12-month FU visit at the time of analysis, unless censored.

Regarding baseline therapy, 66 WWT2D had switched from another GLP-1 RA to oral semaglutide, and 46.9 % were on an SGLT2i. All demographic and baseline characteristics of the WWT2D are shown in Table 1.

### 3.1. Metabolic Control, Weight Loss and Combined Endpoint in Women

**Figure 1:**
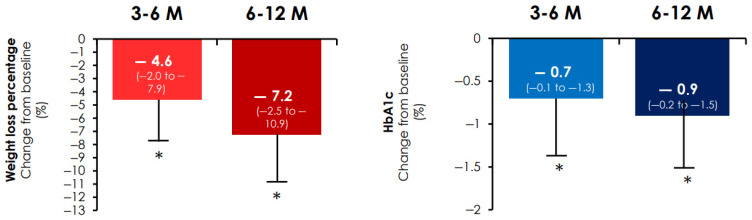
Percentage of weight loss and change in HbA1c from baseline. Percentage of weight loss (**left**) and HbA1c change (**right**) from baseline. * *p* < 0.001 in the Wilcoxon signed-rank test for paired simple changes in weight or HbA1c vs. baseline. HbA1c: glycated haemoglobin; M: month follow-up; Median (interquartile range (IQR); error bars are IQR).

After 6 months of treatment with oral semaglutide, HbA1c decreased by 0.7 points (−0.1 to −1.3 (*p* < 0.001) and weight was reduced by 4.6% (−2.0 to −7.9) (Figure 1) (*p* < 0.001). In addition, a weight loss ≥10% was observed in 13.7% (95% CI −10.8 to −17.0) of WWT2D and systolic blood pressure was reduced by 5 mmHg (−1.0 to −19.5) (*p* < 0.001 for both comparisons).

After 12 months of treatment with oral semaglutide, the reduction in HbA1c was 0.9% (−0.2 to −1.5). While the median weight loss was 7.2% [−2.5 to −10.9], in 29.8% (95% CI −25.8 to −34.1) of WWT2D the weight loss was greater than 10% (Figure 1). The reduction in systolic blood pressure was 5 [−1 to −14.5] mm Hg (*p* < 0.001 for all).

In WWT2D treated with oral semaglutide, the combined endpoint was achieved in 23.5% (95% CI −19.8 to −27.5) of cases in the short term and 29.8% (95% CI −25.9 to −34.1) in the medium term.

At six months, women with a baseline HbA1c ≥ 8% (27.4% vs. 11.8%, *p* = 0.000) or a BMI ≥ 35 kg/m^2^ (25.8% vs. 17.1%, *p* = 0.009) achieved the primary composite endpoint at a higher rate. These differences persisted after 12 months.

**Figure 2:**
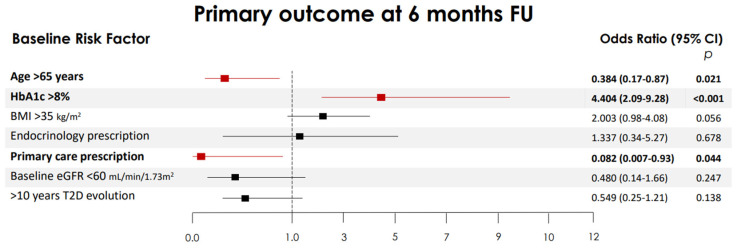
Independent risk factors associated with the achievement of the primary combined endpoint. Multiple logistic regression models constructed to explore independent risk factors associated with the primary combined endpoint at 6 months: defined as a weight loss of ≥5% from the baseline and an HbA1c reduction of ≥1%. The 95% confidence intervals (CIs) of the odds ratios have been adjusted for multiple testing. In bold, independent predictors associated with the outcomes. Variables were included as covariates if they showed significant associations in simple models. BMI, body mass index (BMI); HbA1c, glycated hemoglobin; eGFR, estimated glomerular filtration rate; T2D, type 2 diabetes.

At 6 months, in multivariable analysis, women with a baseline HbA1c ≥ 8% were more likely to reach the primary endpoint [aOR 4.4 (2.1–9.3)] *p* < 0.001, while those with a BMI ≥ 35 kg/m^2^ almost reached statistical significance [adjusted odds ratio, aOR: 2.0 (0.98–4.1)] *p* = 0.05. Age > 65 years [aOR: 0.38 (0.17–0.86)] and a primary care prescription of oral semaglutide [aOR: 0.08 (0.007–0.93)] reduced the odds of achieving the primary outcome (Figure 2). At 12 months, a baseline HbA1c ≥ 8% [aOR: 4.99 (2.33 to 10.73)] and age > 65 years [aOR: 0.41 (0.19 to 0.85)] maintained the association.

### 3.2. Persistence, Reasons for Discontinuation and Safety in Women

Oral semaglutide discontinuation was observed in 185 women [18.2% (95% CI 15.9 to 20.6)]. The most common reasons were lack of continuity of care [renewal/titration; 4.4% (95% CI 3.3 to 5.8)] and gastrointestinal intolerance [9.8% (95% CI 8.1 to 11.8)]. In multivariate analysis, no factors were associated with adherence to oral semaglutide.

No serious adverse events, including severe hypoglycaemia or acute pancreatitis, were observed in women.

### 3.3. Differences by Biological Sex

**Figure 3:**
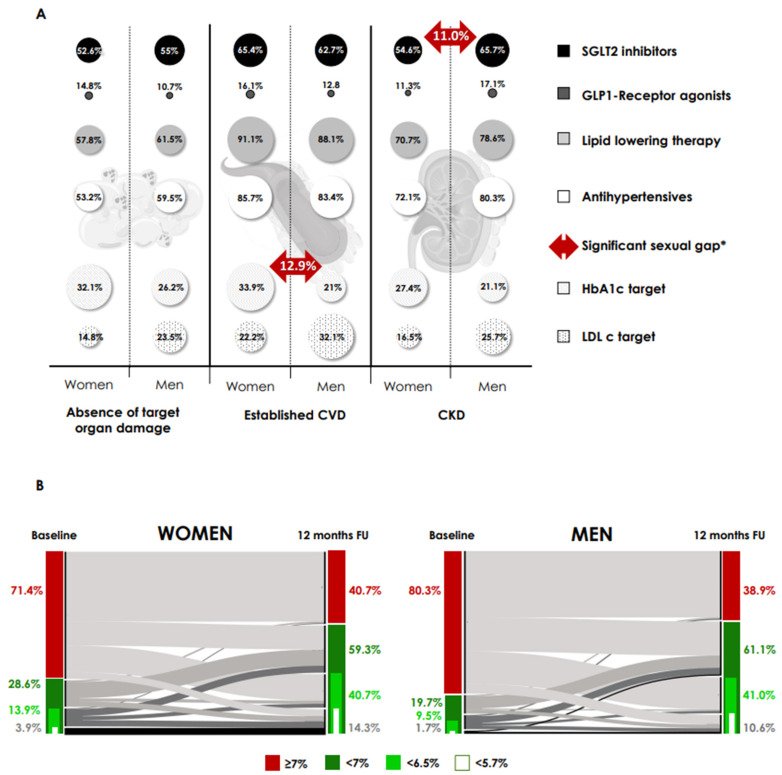
Sex differences in use of cardio-reno-metabolic therapy and compliance with targets at baseline and proportion of patients achieving HbA1c at follow-up. (**A**) Comparative baseline use of drugs in the cardio-reno-metabolic spectrum, and compliance with metabolic and lipid control targets, according to comorbidities. Absence of target organ damage: absence of CVD or CKD. Established CVD: ischaemic heart disease, peripheral arterial disease and cerebral vascular disease. CKD, chronic kidney disease; CVD, cardiovascular disease; HbA1c target, HbA1c < 7%; LDLc, LDL cholesterol < 70 mg/dL and <55 mg/dL, in primary and established CVD/CKD, respectively. * *p* = 0.04 for all (McNemar’s test-differences). (**B**) Alluvial plot of change in HbA1c from baseline at 12-month FU and proportion of patients achieving HbA1c categories at FU by sex (women (right) and men (left) with type 2 diabetes that undergoing treatment with oral semaglutide). Only subjects with HbA1c data recorded at this time point were included. For all, *p* < 0.001 in the McNemar’s test-differences in the proportion of patients achieving HbA1c categories compared to baseline. There were no differences between women and men in the proportion of patients achieving HbA1c targets. FU, follow-up.

Baseline characteristics by sex are shown in Table 2, while comparative baseline use of medications across the cardio-reno-metabolic spectrum and compliance with metabolic and lipid control targets according to comorbidities are shown in Figure 3A.

Overall, women had lower baseline usage of SGLT2i (50.2% vs. 59.9%, *p* < 0.001), statins (61% vs. 69.2%, *p* = 0.005) and antihypertensives (59% vs. 69.8%, *p* < 0.001). However, this difference disappeared after stratification for comorbidities (absence of target organ damage, cardiovascular or chronic kidney disease). Subsequently, the only sex-related difference in baseline treatment was a lower baseline use of SGLT2i in women with CKD (54.6% vs. 65.7%, *p* = 0.04). Women with established cardiovascular disease had better metabolic control (33.9% vs. 21.0%, *p* = 0.04). There were no differences in median semaglutide dose between women and men at the 6- and 12-month FU visits (9.17 vs. 9.19 mg, 11.44 vs. 11.78 mg, *p* > 0.1 for all).

We analyzed the achievement of glycaemic targets on FU in women and men (Figure 3B). Only at the 6-month FU visit, we observed differences between women and men in the goal of HbA1c < 6.5% (40.1% women vs. 32.5% men, *p* = 0.03). At 12 months, there were no differences in the percentage of patients achieving HbA1c targets between women and men. The percentages of women below 7% and 6.5% were 59.3% and 40.7%, respectively, while the percentages of men below these thresholds were 61.1% and 41.0%, respectively.

No differences in weight loss between women and men were observed: −4.4% (−7.5 to −2.0) vs. −4.5% (−7.8 to −2.0), *p* = 0.85 at 6 months; −6.9% (−10.8 to −3.2) vs. −6.8% (−10.7 to −3.2), *p* = 0.73, at 12 months). Also, discontinuation rate (23.7% vs. 22.7%, *p* = 0.710) and reported adverse events (2.5% vs. 1.4%, *p* = 0.354) were similar.

## 4. Discussion

In this real-world study, we show that oral semaglutide is effective and safe in WWT2D. Thus, we report a significant improvement in glycaemic control, and a median weight loss of 7% (with more than 10% weight loss in almost a third of cases), despite low use of the highest dose of oral semaglutide. In addition, the combined medium-term endpoint of weight loss of more than 5% and a one-point reduction in HbA1c was achieved in almost one-third of patients.

Worse baseline metabolic control was associated with achieving the primary endpoint, while older age and prescription of oral semaglutide by Primary Care reduced the odds. At baseline, WWT2D with chronic kidney disease had a lower use of SGLT2i but better metabolic control in secondary cardiovascular prevention, whereas the metabolic and weight loss efficacy, safety and persistence of oral semaglutide were not influenced by sex.

Sex differences in response to oral semaglutide have been largely unexplored. WWT2D have a higher risk of developing cardiovascular disease (CVD) and heart failure (HF) than men with T2D [1], making treatment with antidiabetic therapies with proven cardiovascular and renal benefits mandatory. However, women are typically under-represented in clinical trials [3], which means that the efficacy and safety of current CVD treatments in women are still poorly understood. Real-world evidence can validly complement the essential evidence on drugs that we obtain from RCTs by filling existing gender gaps on their efficacy and safety in clinical practice. Our results support the benefit and safety of oral semaglutide in maintaining tight glycaemic control in WWT2D, which may help to bridge the sex gap in T2D treatment.

The effect of sex on the response to GLP-1 RAs is controversial. Previous studies have suggested that sex may be a predictor of glycaemic improvement following initiation of GLP-1 RAs treatment, but other authors have not found such an association, so a definitive conclusion has not yet been drawn [5,9,11]. Although we observed baseline differences in median HbA1c and target achievement by sex, these differences disappeared in those who remained on oral semaglutide. Even though Cardoso et al. [9] showed a greater predicted HbA1c benefit with GLP-1 RAs in WWT2D, semaglutide (oral or subcutaneous) was not included in the evaluation in either observational studies or clinical trials. Again, the pharmacokinetic data showing higher circulating GLP-1 RA drug concentrations and, consequently, greater HbA1c reductions in women compared with male participants were limited to lixisenatide [12].

We found no differences in weight loss between women and men treated with oral semaglutide. Previous studies have found that women achieve greater weight loss when treated with GLP-1 RAs, although other authors have not shown differences [6,10,13,14]. Differences in GLP-1 RAs evaluated may explain differences in results [11,14]. In terms of factors associated with the achievement of the combined primary endpoint in WWT2D, the association between poorer metabolic control and greater response is well established [15]. Older women may be less active or can have more comorbidities that affect exercise capacity, explaining the reduced likelihood of a better clinical response. The negative impact of primary care prescribing may indicate less familiarity with GLP-1 RAs, which may affect management of side effects and dose optimization.

The discontinuation rate in WWT2D women was like that observed in the whole population in our previous study [10] and is consistent with the results of Piccini et al. in WWT2D in primary prevention [16]. These data have two implications: first, the higher incidence of GI side effects in women treated with GLP-1 RAs may not be true, at least for oral semaglutide; second, there is a huge ceiling for improvement in persistence with GLP-1 RAs in clinical practice. In this context, the person-centered discussion, including cardiovascular risk in diabetes and the benefits demonstrated with GLP-1 RAs, is essential [17].

Our data do not support the notion that WWT2D are less likely than men to receive guideline-recommended CVD treatments [2,16]. This discrepancy may be explained by the clinical setting in a public health system and the differential assessment of the use of target organ-protective therapies according to comorbidities. We found a lower use of SGLT2i only in WWT2D and chronic kidney disease. This suggests that there is room for improvement in addressing gender disparities in renal protection. It is known that WWT2D with diabetic kidney disease have milder albuminuria than men [18], as in our findings, which could be one of the reasons for this lower use of nephroprotective therapies.

Our study has certain limitations but also some important strengths. The limitations include the utilization of digital medical records, which may have certain inaccuracies or missing information. In addition, the lack of consideration of medication-taking behavior may limit the interpretation of our results, as this is a key aspect of clinical decision making regarding the route of administration of semaglutide. The interpretation of comparative effectiveness between men and women could be limited by baseline differences in comorbidities and metabolic control. Finally, this is a post hoc analysis of the original study and may carry some limitations inherent to this design. On the other hand, strengths of this study include the first real-world evaluation of the clinical effectiveness of oral semaglutide in WWT2D, the large sample included, the multicentre and nation-wide design of the study, and the analysis of factors associated with clinical response and persistence in this population.

## 5. Conclusions

In conclusion, oral semaglutide was effective and safe in WWT2D in this real-world study, with nearly one-third reporting weight loss > 10% and more than two-thirds achieving HbA1c < 7%. The effectiveness and persistence of oral semaglutide were not affected by biological sex and there were no safety concerns. Our findings may help to address sex differences in the treatment of T2D.

## Figures and Tables

**Table 1:** Demographic and baseline characteristics of women with type 2 diabetes.

Demographic Characteristics	
Age, years	63 (56–71)
**Clinical characteristics**	
Body weight, kg	
BMI, kg/m^2^	34.4 (31.2–40.1)
<30 kg/m^2^, n (%)	26 (5.5)
30–35 kg/m^2^, n (%)	177 (37.7)
35–40 kg/m^2^, n (%)	86 (18.3)
≥40 kg/m^2^, n (%)	100 (21.3)
Diabetes duration, years	7 (2–13)
**Biochemical parameters**	
HbA1c, %	7.7 (6.8–8.8)
eGFR, mL/min/1.73 m^2^	89 (73–90)
**Comorbidities**	
Ischemic heart disease, n (%)	42 (9)
Stroke, n (%)	21 (4.5)
Peripheral arteriopathy, n (%)	25 (5.5)
Heart failure, n (%)	31 (6.6)
Diabetic kidney disease, n (%)	83 (17.7)
Diabetic retinopathy, n (%)	45 (9.6)
Diabetic neuropathy, n (%)	21 (4.5)
**Antidiabetic medication**	
Naïve, n (%)	27 (5.8)
SGLT-2i, n (%)	220 (46.9)
Biguanides, n (%)	363 (77.4)
DPP-4i, n (%)	151 (32.2)
Secretagogues, n (%)	45 (9.5)
Any GLP-1 RAs, n (%)	66 (14.1)
Liraglutide, n (%)	9 (1.9)
Dulaglutide, n (%)	17 (3.6)
Subcutaneous semaglutide, n (%)	43 (9.2)
Any insulin, n (%)	124 (26.4)
Basal insulin, n (%)	82 (17.5)
**Main medical specialties prescribing oral semaglutide**	
Endocrinology, n (%)	297 (63.3)
Primary care, n (%)	121 (25.8)
Cardiology, n (%)	18 (3.8)
**Highest oral semaglutide dose**	
6-month FU visit, n (%)	274 (34.4)
12-month FU visit, n (%)	564 (67.7)

Values are presented as (%) for categorical variables, and as median (interquartile range, Q1–Q3) for those continuous variables without a normal distribution. BMI, body mass index; HbA1c, glycated haemoglobin; eGFR, estimated glomerular filtration rate; SGLT2-i, sodium-glucose cotransporter 2 inhibitor; DPP-4-i, dipeptidyl peptidase-4 inhibitor; GLP-1RAs, glucagon-like peptide-1 receptor agonist; FU, follow-up.

**Table 2:** Sex-specific differences in risk factors and clinical features of women and men with type 2 diabetes.

		Women (N = 469)	Men (N = 551)	*p*
Age (median; IQR), years		63 (56–71)	63 (56–70)	0.841
	>65	209 (44.9%)	241 (44.3%)	0.837
	>75	82 (17.6%)	62 (11.4%)	**0.005**
Ischemic heart disease (%)	No	427 (91%)	402 (73%)	**<0.001**
	Yes	42 (9%)	149 (27%)	
Stroke (%)	No	392 (92.7%)	450 (89.8%)	0.129
	Yes	31 (7.3%)	51 (10.2%)	
Stroke (%)	No	448 (95.5%)	509 (92.4%)	**0.038**
	Yes	21 (4.5%)	42 (7.65%)	
Peripheral artery disease (%)	No	444 (94.7%)	492 (89.3%)	**0.002**
	Yes	25 (5.3%)	59 (10.7%)	
Diabetic kidney disease (%)	No	385 (82.3%)	392 (71.1%)	**<0.001**
	Yes	83 (17.7%)	159 (28.9%)	
Diabetic retinopathy (%)	No	424 (90.4%)	464 (84.4%)	**0.004**
	Yes	45 (9.6%)	86 (15.6%)	
Diabetic neuropathy (%)	No	448 (95.5%)	510 (92.7%)	0.061
	Yes	21 (4.5%)	40 (7.3%)	
GLP1 RA (%)	No	398 (85.2%)	481 (87.5%)	0.301
	Yes	69 (14.8%)	69 (12.5%)	
Insulin (%)	No	331 (72.6%)	380 (71.8%)	0.792
	Yes	125 (27.4%)	149 (28.2%)	
SGLT2i (%)	No	227 (49.8%)	212 (40.1%)	**0.002**
	Yes	229 (50.2%)	317 (59.9%)	
Metformin (%)	No	105 (23.1%)	104 (19.7%)	0.185
	Yes	349 (76.9%)	425 (80.3%)	
Change from iDPP4 (%)	No	305 (67.5%)	351 (66.7%)	0.804
	Yes	147 (32.5%)	175 (33.3%	
Statins (%)	No	180 (38.8%)	167 (30.8%)	**0.008**
	Yes	284 (61.2%)	375 (69.2%)	
Hypertension drugs (%)	No	185 (40.7%)	163 (30.2%)	**0.001**
	Yes	270 (59.3%)	376 (69.8%)	
LDLc (median; IQR), mg/dL		118.3 (73.8–118.3)	81.5 (58–109)	**<0.001**
Triglycerides (median; IQR), mg/dL		156.5 (112–215)	165 (118–250)	**0.019**
Platelets (median; IQR), mcL		263 (215–301.3)	228 (186–264)	**<0.001**
AST (median; IQR), U/L		20 (16–29)	22 (17–32.3)	**0.001**
ALT (median; IQR), U/L		21 (16–33)	26 (18–40)	**<0.001**
FIB-4 (median; IQR)		1.11 (0.81–1.53)	1.35 (0.91–1.86)	**<0.001**
	F0–1	180 (63.2%)	166 (47.8%)	**<0.001**
	F2	95 (33.3%)	169 (48.7%)	**<0.001**
	F3–4	10 (3.5%)	12 (3.5%)	0.972
	<1.3	180 (63.2%)	166 (47.8%)	**<0.001**
	≥1.3	105 (36.8%)	181 (52.2%)	
Diabetes duration (median; IQR), years		7 (2–13)	8 (3–12)	0.225
oSEMA doce (mg)	3	431 (92.1%)	501 (90.9%)	0.611
	7	17 (3.6%)	27 (4.9%)	
	14	20 (4.3%)	23 (4–2%)	
eGFR (median; IQR), mL/min/m^2^		89 (73–90)	87 (63.3–90)	**0.027**
	≥60 mL/min/1.73 m^2^	396 (86.3%)	425 (78.6%)	**0.002**
	<60 mL/min/1.73 m^2^	63 (13.7%)	116 (21.4%)	
ACR (median; IQR), mg/g		9 (3–30)	15 (5–58.8)	**<0.001**
	CAC > 30	67 (24.5%)	123 (35.8%)	**0.002**
	CAC > 300	15 (5.5%)	26 (7.6%)	0.301
CKD (%)	No	178 (58.9%)	182 (46.2%)	**0.001**
	Sí	124 (41.1%)	212 (53.8%)	
HbA1c (median; IQR), mmol/mol		7.6 (6.8–8.6)	8 (7–8.8)	**0.002**
Height (median; IQR), m		1.59 (1.54–1.68)	1.70 (1.66–1.75)	**<0.001**
Weight (median; IQR), kg		88.95 (79.85–102)	98.5 (89–112)	**<0.001**
BMI (median; IQR), kg/m^2^		34.6 (31.2–40)	33.4 (31.1–37.8)	**0.006**
	IMC > 27.5	380 (97.7%)	429 (97.9%)	0.799
SBP (median; IQR), mmHg		136 (125–148)	139 (126–149)	0.463
DBP (median; IQR), mmHg		80.5 (74–88)	82 (74–90)	0.287

IQR: interquartile range, LDLc: low-density lipoprotein cholesterol., AST: aspartate aminotransferase, ALT: alanine aminotransferase, oSEMA: oral semaglutide, eGFR: glomerular filtration rate, ACR: albumin–creatinine ratio, CKD: chronic kidney disease, HbA1c: glycosylated hemoglobin, m: meters, kg: kilograms, BMI: body mass index. In bold, significant differences.

## Data Availability

The data presented in this study are available on request from the corresponding author due to ethical restriction.
